# Contralateral Acupuncture for the Treatment of Phantom Limb Pain and Phantom Limb Sensation in Oncologic Lower Limb Amputee: A Case Report

**DOI:** 10.3389/fnins.2021.713548

**Published:** 2021-10-22

**Authors:** Qin Guo, Zhong Di, Hong-fang Tian, Quan-ai Zhang

**Affiliations:** Department of Acupuncture and Moxibustion, The Third Affiliated Hospital of Zhejiang Chinese Medical University, Hangzhou, China

**Keywords:** acupuncture, contralateral acupuncture, phantom limb pain (PLP), phantom limb sensation (PLS), phantom limb

## Abstract

Phantom limb pain (PLP) and phantom limb sensation (PLS) are common and distressing sequelae of amputation. Current pain management following amputation is challenging and unsatisfying. In this case study, a 74-year-old woman underwent above-knee amputation because of the rhabdomyosarcoma in the right leg. Despite several analgesics, pain was poorly controlled. The phantom limb pain and sensation were immediately reduced by the contralateral acupuncture, and abolished after the third session with no side-effects, no relapse during the next 9 months. Contralateral acupuncture showed positive effect on PLP and PLS in this case, but more robust evidence would be needed to support the efficacy of this treatment technique for indication.

## Introduction

Most amputees complain of various distressing sensations in the amputated limb such as burning, stinging, shooting, piercing or cramping pain, known as phantom limb pain (PLP), and non-painful phantom limb sensations (PLS), (e.g., temperature, pressure, itching, touch), (Stankevicius et al., 2021). The lifetime prevalence of PLP and PLS are 76–87 and 87% respectively (Stankevicius et al., 2021). Phantom limb pain may occur immediately or in the following days after surgery, and may gradually diminish over a few months to decades (Raggi and Ferri, 2019; Erlenwein et al., 2021). These may affect all aspects of life, including mood, sleep, family relationship, and social interaction (Colquhoun et al., 2019) which in turn will aggravate the intractable sensations and form a vicious circle.

The underlying etiopathology of PLP is complex including peripheral, spinal, and brain mechanisms (Kaur and Guan, 2018). Commonly used treatments for PLP include pharmacotherapy (analgesics, anesthetics, muscle relaxants, antidepressants, and anticonvulsants, etc) and non-pharmacological interventions (transcutaneous electrical nerve stimulation, mirror therapy, virtual reality, and acupuncture, etc), (Erlenwein et al., 2021). Disappointingly, none of these interventions has been proved to be consistently effective (Aternali and Katz, 2019).

Acupuncture treatment for PLP or PLS has been documented in the literature (Davies, 2013; Mannix et al., 2013; Trevelyan et al., 2016). Here, we report a PLP and PLS case post-amputation with good response to acupuncture.

## Case Report

**Figure 1:**
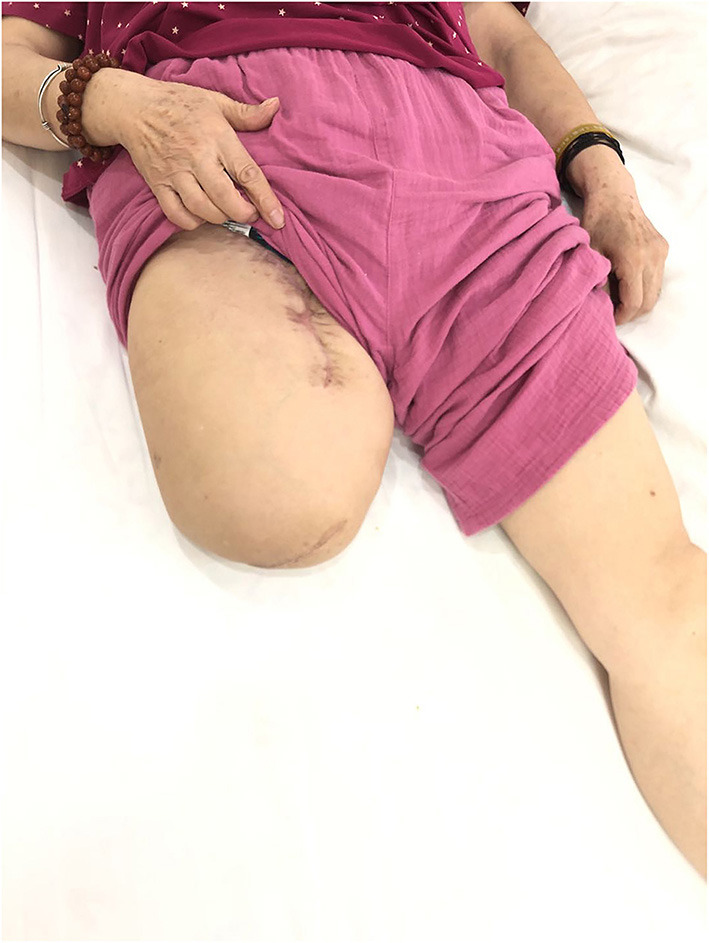
The patient's condition after right thigh amputation and inguinal lymph node dissection.

In August 2018, a 74-year-old female underwent resection of the right calf mass, followed by chemotherapy and local radiotherapy. Post-operative pathology reported: right calf spindle cell malignant tumor, rhabdomyosarcoma. After surgery, rhabdomyosarcoma of the right leg recurred and metastasized to superficial inguinal lymph nodes, so thigh amputation and inguinal lymph node dissection were performed under remifentanil-propofol anesthesia on June 8, 2020. Routine antibiotics, analgesia, dressing change and symptomatic support were used after operation. The PLP could not be well-managed by several analgesics, including dezocine injection, flurbiprofen axetil injection, parecoxib sodium for injection, loxoprofen sodium tablets, diclofenac sodium sustained release tablets and tramadol hydrochloride tablets. With a glimmer of hope, she came to our hospital for palliation on July 17, 2020. Upon admission, most of the incisions healed well (Figure 1), pus exuded in the groin, the residual limb was obviously swollen, and the skin temperature was slightly higher. The patient felt that the right lower limb still existed, so that she fell down several times after getting out of bed. Sometimes she felt pressure or itching sensations along her right leg. And she felt burning and stinging pain in the amputated limb, especially around right ankle. The pain intensified at night, so she complained of disturbed sleep and depressed after the amputation.

**Figure 2:**
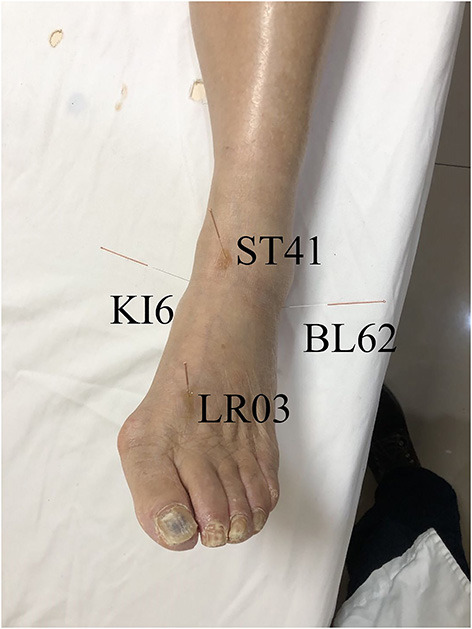
The selected acupoints Shenmai (BL62), Jiexi (ST41), Zhaohai (KI6) and Taichong (LR03) on the left side.

In addition to long-term oral medications such as anlotinib hydrochloride capsules, mecobalamin tablets, vitamin B6 tablets and folic acid tablets, acupuncturist with more than 10 years of work experience arranged acupuncture treatment for her, and no analgesics were taken. Shenmai (BL62), Jiexi (ST41), Zhaohai (KI6) and Taichong (LR03) on the left side were needled by stainless steel acupuncture needles (diameter 0.18 mm; length 13 mm; Suzhou Medical Appliance Factory, China). The needles were twirled after insertion and every 10 minutes thereafter to achieve *de qi* (heaviness, soreness, distension, or numbness sensation typically generated by the insertion or manipulation of the needle), and retained for 30 minutes (Figure 2).

The Neuropathic Pain Symptom Inventory (NPSI), as one of the most widely used scales for characterizing neuropathic pain (Bouhassira et al., 2004), was adopted to measure the patients' PLP and PLS. The pain was long-lasting, but aggravated during the night and disturbed her sleep. The NPSI was measured during the day and night before and after each treatment. Before the treatment, the NPSI was rated 3/10 during the day and 5/10 at night. After the first session, the NPSI immediately dropped to zero, but rebounded to three at night. Then, the patient received another two sessions in the next 2 days. Satisfactorily, the NPSI plunged to zero at all times of the day or night. During the whole sessions, no side-effects were reported. So far, there has been a 9-month follow-up period, and the patient has not suffered a relapse. From the perspective of the patient, she was very satisfied with the treatment, because the PLP and PLS disappeared completely, and her sleep and mood returned to normal in the long term.

## Discussion

In the present case, conventional analgesics did not manage the phantom limb syndrome well. After three sessions of acupuncture treatment without analgesics, the patient achieved a long-term pain relief successfully with free of both phantom limb pain and phantom limb sensation, and improved quality of life. Indubitably, the effectiveness of acupuncture in the treatment of phantom limb pain or phantom limb sensation is questioned.

Through converging evidence, phantom limb pain is classified as a kind of neuropathic pain, and is induced by a lesion of the somatosensory nervous system (Erlenwein et al., 2021). The mechanisms were elucidated at the peripheral level, spinal cord level, the brain and psychological factors (Flor, 2002; Erlenwein et al., 2021). At the periphery level, the injured nerve initiates regenerative processes with abnormal spontaneous activity generating afferent input, followed by an increased expression of sodium channels, higher activity of nociceptive C fibers and spontaneous activity of dorsal root ganglion neurons (Black et al., 2008; Erlenwein et al., 2021). In the spinal cord, sensitization is present with reduced inhibitory interneurons, increased spinal pro nociceptive excitatory systems, increased glutamate and the N-methyl-D-aspartate (NMDA) receptor system (Nikolajsen et al., 1996; Flor, 2002; Erlenwein et al., 2021). In the brain, cortical reorganization and neuronal plasticity of the somatosensory cortex are indicated after amputation (Flor et al., 2006). Particularly, imaging studies have reported cortical remapping in amputees (Cruz et al., 2003). Psychological factors such as depression and anxiety may affect the course and the severity of phantom limb pain (Fuchs et al., 2018; Erlenwein et al., 2021). Due to the multiplex mechanisms underlying phantom limb pain, conventional treatment, typically targets a single proposed mechanism, seems to be inadequate (Aternali and Katz, 2019). Therefore, a special therapy that aims at multiple mechanisms of phantom limb pain seems to be optimum, but has not yet been presented.

Acupuncture, as an ancient Chinese medical technique, has been widely applied in the treatment of various types of pain over 3,000 years, and accumulating experimental and clinical studies have demonstrated its benefit for chronic pain during the last decade (Vickers et al., 2018; Gao et al., 2021). Numerous reviews have summarized the analgesic effects of acupuncture regarding the peripheral, spinal, and supraspinal mechanisms (Zhang et al., 2014; Lai et al., 2019; Lyu et al., 2021). Several studies indicate that peripheral opioids, induced by acupuncture, act on peripheral opioid receptors to desensitize peripheral sensory nerves and reduce pro-inflammatory cytokines peripherally (Zhang et al., 2014). Acupuncture inhibits the transmission of noxious inputs in the spinal cord with the involvement of spinal opioids, norepinephrine, serotonin, glutamate and glial cell (Zhang et al., 2014). Acupuncture analgesia is associated with downregulation of glutamate in the ascending excitatory pathway and upregulation of opioids, norepinephrine, and 5-hydroxytryptamine in the descending pain modulatory system, thus contributing to alleviate central sensitization (Lyu et al., 2021). From another perspective, affective disorders are interrelated with pain (Becker et al., 2018), and somatosensory pain memories may be revived after an amputation and lead to phantom limb pain (Katz and Melzack, 1990). Acupuncture can improve pain-related mood disorders (Shi et al., 2020) and alleviate retrieval of pain memory (Sun et al., 2015). In a word, acupuncture can manage the multidimensional nature of pain with the capability of restoring homeostasis, (Li et al., 2017; Lin et al., 2020) which seems to be an optimal adjuvant therapy (Tseng et al., 2014). More importantly, this complies with the holistic concept of traditional Chinese medicine, because Chinese medicine believes that the human body is an organic whole. In this report, amputation caused a series of imbalances, resulting in PLP and PLS, and contralateral acupuncture cured the PLP and PLS, which may be related to the restoration of the entire imbalance.

Several case reports and a randomized controlled study have documented positive outcome on acupuncture Treatment of phantom limb pain or phantom limb sensation (Bradbrook, 2004; Jacobs and Niemtzow, 2011; Davies, 2013; Tseng et al., 2014; Trevelyan et al., 2016). A systematic review has concluded that acupuncture therapy has a positive effect on the symptoms of phantom-limb syndrome (Mannix et al., 2013). Because case series are at a low level in the evidence hierarchy, and randomized controlled trials are rare, there is insufficient evidence to support the use of acupuncture for phantom limb pain or phantom limb sensation. Among these case reports, the style of acupuncture and choice of acupoints are quite different (Bradbrook, 2004; Jacobs and Niemtzow, 2011; Davies, 2013; Mannix et al., 2013; Tseng et al., 2014; Trevelyan et al., 2016). Because acupuncture requires different methods and acupoints for different conditions, it is difficult to form a standard protocol. In this case, the patient felt pain especially around ankle, therefore commonly used acupoints around ankle were chosen, namely Shenmai (BL62), Jiexi (ST41), Zhaohai (KI6) and Taichong (LR03).

The type of acupuncture we chose for this patient is contralateral acupuncture, which is originally recorded in an ancient classic of Chinese medicine, with the title of the *Huang Di Nei Jing* (*The Yellow Emperor's Canon of Internal Medicine*). Contralateral acupuncture means inserting needle on the side opposite the disease side, and is widely used in various diseases with good response, such as PLP, chronic shoulder pain, post-herpetic neuralgia, episodic cluster headache, acute traumatic pain, apoplectic hemiplegia, hemihidrosis, dizziness (Cheng, 1996; Lu, 1997; Sui and Huang, 2004; Davies, 2013; Hayhoe, 2016; Zhang et al., 2016). The mechanism of contralateral acupuncture to treat pain involves both peripheral and central nerve systems, including spinal interneurons, endogenous opioids and diffuse noxious inhibitory controls (DNIC), (Lianfang, 1987; Bing et al., 1990, 1991). For example, study showed contralateral acupuncture produced analgesic effects by directly modulating the anterior cingulate cortex (ACC) and other brain areas, and lesions of rostral ACC completely abolished the anti-nociceptive effects of contra-but not ipsi-lateral acupuncture (Yi et al., 2011). However, the specific mechanism of contralateral acupuncture for phantom limb pain or sensation remains unclear.

## Conclusion

In conclusion, our case demonstrates that contralateral acupuncture abolishes the phantom limb pain and phantom limb sensation, and improves the quality of life in the amputee of tumor-bearing lower limb. Based on our experience, contralateral acupuncture may be an efficacious, economical and safe adjunct, but further research with large samples is required to probe the mechanism and efficacy.

## Data Availability Statement

The original contributions presented in the study are included in the article/supplementary material, further inquiries can be directed to the corresponding author.

## Ethics Statement

Ethical review and approval was not required for the study on human participants in accordance with the local legislation and institutional requirements. The patients/participants provided their written informed consent to participate in this study. Written informed consent was obtained from the individual(s) for the publication of any potentially identifiable images or data included in this article.

## Author Contributions

Q-aZ contributed to the conception and design of the study. ZD contributed to the treatment and analysis of data. QG contributed to drafting the text and preparing the figures. All authors read and approved the manuscript.

## Funding

This work was supported by the National Innovative and Core Talents Project of Traditional Chinese Medicine (grant number ZYYCY201901) and National Natural Science Foundation of China (grant number 82004463).

## Conflict of Interest

The authors declare that the research was conducted in the absence of any commercial or financial relationships that could be construed as a potential conflict of interest.

## Publisher's Note

All claims expressed in this article are solely those of the authors and do not necessarily represent those of their affiliated organizations, or those of the publisher, the editors and the reviewers. Any product that may be evaluated in this article, or claim that may be made by its manufacturer, is not guaranteed or endorsed by the publisher.
